# Developing health science students into integrated health professionals: a practical tool for learning

**DOI:** 10.1186/1472-6920-7-45

**Published:** 2007-11-15

**Authors:** Lorna Olckers, Trevor J Gibbs, Madeleine Duncan

**Affiliations:** 1 School of Public Health and Family Medicine, Faculty of Health Sciences, University of Cape Town, Cape Town, South Africa; 2 Bute Medical School, University of St Andrews, St Andrews, Scotland, UK; 3 School of Health and Rehabilitation Sciences, Faculty of Health Sciences, University of Cape Town, Cape Town, South Africa

## Abstract

**Background:**

An integrated sense of professionalism enables health professionals to draw on relevant knowledge in context and to apply a set of professional responsibilities and ethical principles in the midst of changing work environments [1,2]. Inculcating professionalism is therefore a critical goal of health professional education. Two multi-professional courses for first year Health Science students at the University of Cape Town, South Africa aim to lay the foundation for becoming an integrated health professional [3]. In these courses a diagram depicting the domains of the integrated health professional is used to focus the content of small group experiential exercises towards an appreciation of professionalism. The diagram serves as an organising framework for conceptualising an emerging professional identity and for directing learning towards the domains of 'self as professional' [4,5].

**Objective:**

This paper describes how a diagrammatic representation of the core elements of an integrated health professional is used as a template for framing course content and for organising student learning. Based on the assumption that all health care professionals should be knowledgeable, empathic and reflective, the diagram provides students and educators with a visual tool for investigating the subjective and objective dimensions of professionalism. The use of the diagram as an integrating point of reference for individual and small group learning is described and substantiated with relevant literature.

**Conclusion:**

The authors have applied the diagram with positive impact for the past six years with students and educators reporting that "it just makes sense". The article includes plans for formal evaluation. Evaluation to date is based on preliminary, informal feedback on the value of the diagram as a tool for capturing the domains of professionalism at an early stage in the undergraduate education of health professional students.

## Background

The primary health care approach (PHC), which was adopted by the government of national unity following the demise of apartheid in South Africa in 1994, advocates the transformation of the health services and its workers within available socio-economic infrastructures necessary to attain health for all [5-8]. The Alma Ata Declaration [9], which forms the philosophical basis of the PHC approach, suggests that *"...primary health care relies, at local and referral levels, on health workers who are suitably trained – socially and technically – to work as a health team and to respond to the expressed health needs of the community"*. Socially responsive health professional education is therefore concerned with developing practitioners who, through their professionalism and competence, make a contribution not only to the health needs of individuals but also to community development [10]. Working towards shared service objectives means that health professional graduates require profession specific as well as generic skills for interpreting and addressing the health needs of individuals, groups and communities. Learning about professionalism provides a generic foundation on which health science students can build a shared appreciation of the intrapersonal, interpersonal and public dimensions of their professional behaviour, parameters and responsibilities [11,12].

### Generic educational outcomes

Competence in health promotion strategies, in the prevention of ill-health and in the application of curative interventions and rehabilitation technologies, students should also be able to work with social responsiveness in mind [13]. In South Africa, comprehensive healthcare requires students to collaborate with a diverse range of health workers and other role players such as traditional healers, community representatives, rehabilitation assistants and consumers [14]. Students need to be politically astute, culturally competent and emotionally mature with a strong professional identity. In short, health profession graduates need to be "integrated". This implies that at undergraduate level, students should have conceptualised and internalised professionalism as affecting their personal 'doing', 'being' and 'becoming'. Professionalism is not an abstract concept 'out there'; it requires active engagement of the knowledge, interpersonal and intrapersonal domains of the evolving 'self as health professional' [14,15]. The iterative, educational process of becoming integrated [16,17] occurs through conceptualising and 'doing' (practicing) the following three dimensions of professionalism as part of the generic course outcomes:

• being knowledgeable (having and continually seeking a sound grasp of the facts and scientific evidence),

• being empathic (continually seeking to understand the 'other')

• being reflective (continually seeking to make sense of experience by critically thinking through personal and interpersonal actions and reactions)

Instigated early in professional training, these educational outcomes lay the foundation on which uni-professional role identity may be built. A shared appreciation of an integrated health professional also fosters commitment amongst future health workers to the principles and values of primary health care and professionalism in particular [4].

### Developing the integrated health professional

Professionalism is associated with respectful self-presentation, a caring attitude, interpersonal competence and commitment to life long learning [10]. It is often addressed in curricula with reference to professional parameters (legislated codes of practice), behaviours (personal presentation and interpersonal relationships) and responsibilities (to self, profession, employer, society, clients) [10,11,18]. At the University of Cape Town's Faculty of Health Sciences, two multi-professional courses "Becoming a Professional" (BP) and "Becoming a Health Professional" (BHP) form part of the core curriculum and are compulsory for all first year medical, physiotherapy, occupational therapy, audiology and speech and language students [3-5]. Both courses use a conceptual diagram for framing students' learning by focussing their attention on the three central qualities of professionalism described previously: knowledge attainment, empathic practice and reflective thought. The IHP diagram is used to frame the dimensions, relevance and dynamics of professionalism as these pertain to each student's personal learning journey, not only in the BP/BHP courses but also across the other courses they take in becoming a professional healthcare practitioner.

### A diagram for learning

**Figure 1:**
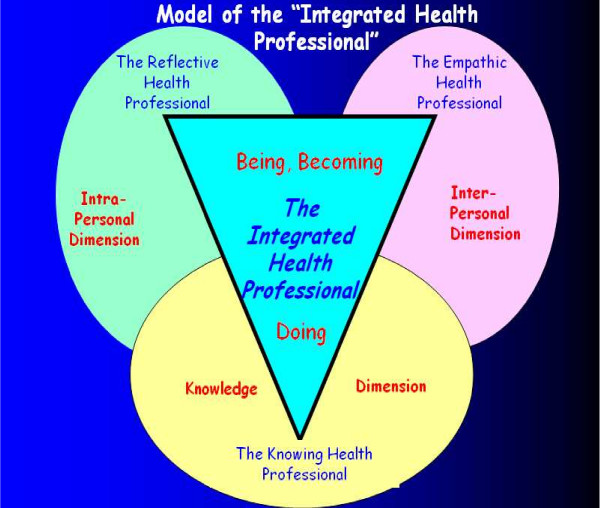
Model of the 'integrated health professional'.

The diagram draws attention to the dynamic interface between the three specific areas of personal-professional development, namely the ***Knowing***, the ***Empathic ***and the ***Reflective ***Integrated Health Professional, and relates to outcomes-based education as it guides students towards discipline specific and meta-appreciation of professionalism [19] (See Figure 1.)

• **The knowing health professional **– relates to the knowledge and technical skill dimensions of professional practice.

• **The empathic health professional **– the development of inter-personal skills based on social understanding and moral-ethical sensitivity

• **The reflective health professional **– the development of the intra-personal dimensions of self-awareness and culturally sensitive attitudes

### The knowing dimension

The **knowing dimension **is related to the concept of life long learning and the acquisition and appropriate application of knowledge. Knowledge is defined as "an awareness or familiarity gained by experience of a fact or situation" [20]. Typically the area of focus for all health professionals, the **knowing dimension **is not just concerned with the acquisition of facts, but also with the development of competencies that enable the professional to practice in increasingly accountable and technological environments [21,22]. The **knowing dimension **includes intraprofessional knowledge but as multi-professionalism grows, it moves from being absolute in its convictions to being responsive to the combined dimensions of the Integrated Health Professional [23]. The **knowing **Integrated Health Professional draws upon prior knowledge to develop expertise and to bring about innovation [24]; uses modern methods of learning [25] and engages emerging understanding to dispel misguided myths regarding the contributions and roles of other professionals and role players. Enacted within a multi-professional learning environment of comparative content [26], the **knowing **professional will also draw upon cultural and social domains of knowledge in order to work towards contextually relevant practice [27].

### The empathic dimension

The **empathic dimension **is concerned with the 'being' and moral-ethical sensitivity of the health professional. Rogers, in his seminal work on client centred care and empathy, suggests that *"...empathy means entering the private world of the other and becoming thoroughly at home in it. It means temporarily living in his/her life, moving about in it delicately without making judgements... as you look with fresh and unfrightened eyes at elements of which the individual is fearful...." *[28] Empathy flows from the interface between the following three forms of understanding within the professional:

• Understanding that comes from professional knowledge and interpretation of information gleaned from research (for example evidence based practice) and investigations (for example profession-specific assessments).

• Understanding that comes from self awareness i.e. the professional is reflective and conscious of how his/her feelings, attitudes and behaviours influence relationships

• Understanding that comes from appreciating the client's frame of reference i.e. by extending positive regard and listening non-judgementally.

Peloquin [29] suggests that empathy is "*finding you in me*". It involves the recognition of likeness; a grasp of the universal nature of human problems; an appreciation of uniqueness and a caring alliance that reveals to 'the other' a belief in their innate capacity to resolve their own issues. Sevenhuijsen [30] in arguing for new practices of professional accountability suggests that the ethics of care cannot be separated from citizenship and the promotion of human rights. Being 'attentive' and empathic promotes a sense of well-being and human dignity because it is based on the moral-ethical principles of beneficence and non-maleficence. 'Caring' and 'helping' are social, moral and political practices that promote human flourishing. To be empathic fulfils the health professional's duty to treat others like he/she would like to be treated as a citizen. This understanding of the **empathic dimension **brings ethics into the relationship between health practitioner and patient/client (individual, group or community) in that it demands 'response-ability' from the professional to the interface between human rights and health [30]. While it is not always feasible or indicated to become involved beyond the client's (group, community) immediate health concerns, the door for empathic exchange needs to be held open as wide as possible in order for social transformation to occur.

### The reflective dimension

The importance of reflective practice has been highlighted in descriptions of socially responsible medical schools [31,32]. The **reflective dimension **is concerned with a process that links the self with the outside, in both a learning (self-enriching) and a design (self-expressing) relationship [33]. The reflective process can often lead to unexpected outcomes since it involves linking feelings, cognition and action in complex interrelated and interactive ways. Negative feelings, especially about the self, can form major barriers to learning. Alternatively, negative feelings can trigger reflexive interrogation of experience that ultimately enhances learning and practice. Positive feelings and emotions can also enhance the learning process because they keep the learner on the task and provide motivation for exploring new interpretations and understanding that may lead to innovations in practice.

Seminal writer Schön [34] suggests that professionals draw on formal knowledge and use "reflection-in-action" when they are working. Barnett [35] describes reflection as "being self-critical". Reflection, in order to be truly meaningful, must be pursued with intent and directed towards the goal of professional excellence while drawing on prior experience. The ultimate guardians of professional excellence are not external forces, but internal professional responsibilities [36]. The **reflective dimension **of the model ensures that attention is paid to both objective and subjective personal learning experiences.

### Unifying features of the diagram

**Figure 2:**
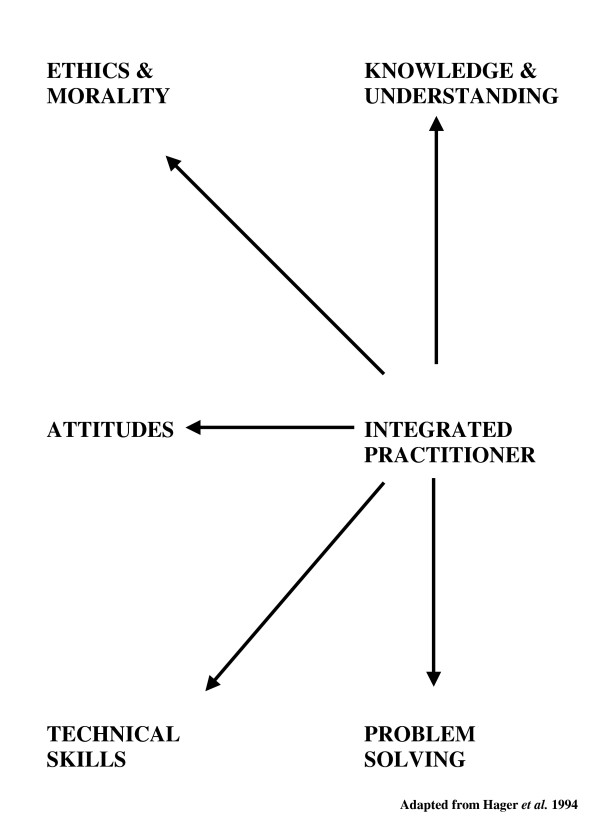
Unifying features of the integrated professional.

The IHP diagram relates to outcomes-based education as it guides students towards discipline specific and meta-appreciation of professionalism [19]. A sense of professional identity emerges as students engage iteratively with being able to do something through gaining knowledge, technical skills and problem solving abilities (**the knowing dimension**), to being able to do it with understanding within the context of self and others (**the empathic dimension**) to being increasingly self aware and culturally sensitive (**the reflective dimension**). The diagram provides students with **a conceptual tool **for learning about professionalism by starting with the self, with focus on the reflective dimension, and moving outwards via small group experiential exercises through which the empathic and knowing dimensions are engaged and developed. Finally, the cycle is completed with a return to the reflective domain in which the student must ask: *"what does this experience mean for me as a future health professional?" *Each iterative cycle of learning interrogates the unifying features of the integrated professional so that students come to recognise that professionalism is dynamic and multifaceted. Figure 2 depicts the unifying features of the integrated professional [37].

The knowing, empathic and reflective domains of the professional are integrated through the dynamic interchange between knowledge, technical skill, understanding, problem solving, attitudes and ethics. The constructive alignment of Figure 1 with Figure 2 in Table 1 provides students and educators with a structure for appreciating the dynamics of professionalism.

**Table 1:** The dynamics of professionalism

Unifying features of the integrated professional	Domains of the integrated professional		
	
	Knowing	Reflective	Empathic
Knowledge	Anticipation of probable experience and outcome based on knowledge about genesis, progression and consequences of health conditions and contexts		
Technical skill	Acquired abilities in addressing identified health and development needs through discipline specific skills		
Understanding	Capacity to base actions on thorough appreciation of the total picture presented by the individual, group or population		
Problem solving	Critical thinking to identify and guide appropriate action in addressing emerging health and development needs		
Ethics	An informed appreciation of the moral-ethical choices that are made by self and others and the reasons behind such choices		
Attitudes	A transformed, culturally relevant way of viewing the world and of thinking that affirms diversity and promotes the ideals of a democratic society.		

### Application of the ihp diagram

In one particular group session during the "Becoming a Professional" (BP) course when learning to actively listening is the core outcome, the small group facilitator asks half of his/her group to go outside the room and instructs them to tell their partners inside the room a story about a local shop keeper, who they must pretend to know, who was brutally attacked. The students sharing the incident must pretend to be shocked and very sad.

The facilitator then tells the inside group that their partners will be telling them a story. They must appear to be listening but act distracted by, for example only occasionally nodding; making poor eye contact; fiddling with their clothing; watching others or laughing at others. The outside group is called in and the role-play exercise begins. Typically what happens is that the group telling the story becomes frustrated and angry by the lack of interest and empathy shown by their partners.

What follows is a debriefing and learning process using the diagram as a tool or point of reference. Students are asked to:

• Reflect on how they felt in the role-play. A student might, for example, say that she found her partner's lack of eye-contact rude and inappropriate making it hard to tell her story. Another student might then comment that direct eye-contact in her community is in itself considered rude and inappropriate, particularly when speaking to older people. Students therefore focus on the reflective dimension through drawing on their more immediate and prior experiences. This process of reflection highlights aspects of ethics and morality and attitude [37].

• Discuss what should have taken place, with students drawing on their own knowledge and experience as well as information from more formal sources such as assigned readings related to listening skills and interviewing. The knowing dimension with emphasis on understanding becomes engaged at this point [37].

• Perform a re-run of the role-play using appropriate responses related to active listening and empathic responses. The technical skills of interpersonal dynamics of the empathic dimension are brought into play through this process.

• Reflect on how it felt to be really listened to and how it felt to listen using the skills learnt in the session.

Learning outcomes are put into practise through the process of acknowledging and 'doing' all three dimensions involved in the process of 'being' and 'becoming' "Integrated Health Professionals". This process ultimately results in understanding of what is really required to be an active listener [37].

Throughout the learning process in the "Becoming a Professional" and "Becoming a Health Professional" courses students are brought back to the "Diagram of the Integrated Health Professional" in order to keep track of their own developing professionalism. A practical example is where facilitators ask students to draw the basic diagram of the Integrated Health Professional for themselves and to then indicate on the diagram how they see their own development at that point in the course. Facilitators then ask students to share this information and to identify specific goals for themselves such as the need to work on the knowledge dimension through gaining further knowledge on the theory of interviewing, or the need to work on the empathic dimension through practising interviewing. The exercise is repeated at strategic evaluation points throughout both courses. In this way the Diagram of the Integrated Health Professional becomes a tool for emerging professionalism, a point of reference for interrogation of student professional development as well as an organising framework for inculcating professionalism.

### Evaluation

The efficacy of the IHP diagram as a tool for learning has yet to be formally evaluated. Students and group facilitators have in their feedback to date been overwhelmingly positive about its effectiveness as a visual tool for guiding learning; a structure for monitoring development; a means for assisting students with becoming more conscious of the importance of their own thoughts, feelings and behaviour, and a way to constantly emphasise the importance of all three dimensions in making sense of complex environments.

A formal evaluation is planned for the end of 2008, thus allowing for several iterations of each course, a significant number of participants and a time element to allow reflection. The evaluation will take the form of a qualitative study in two stages. Final year students will be asked to join focus groups to look back on their own emerging professionalism and to critically evaluate the usefulness of the diagram of the Integrated Health Professional as a tool for understanding and practicing professionalism. The second stage will involve the small group facilitators who have used the diagram as an educational tool in their interactions with students. They too will be asked to participate in focus groups to evaluate the usefulness of the diagram as an educational tool. The results of this evaluation process will be the subject of a future paper.

## Conclusion

The Integrated Health Professional diagram is an innovative, dynamic and multi-layered visual and conceptual tool that can help frame students' development as professionals within their environments of learning. The three dimensions of the this diagram are clearly delineated and deliberately equal in size so that students understand that the Integrated Health Professional focuses equally on all three areas of professionalism. Implicit in the diagram is the need for health professionals to be comfortable and competent in the dimensions of knowledge; empathic interpersonal skills and reflective practise. The efficacy of this tool will be formally evaluated at the end of 2008. Informal feedback from facilitators and students alike has to date been overwhelmingly positive.

As one facilitator commented: *"It is easy to explain and apply ...it just makes sense"*.

## Competing interests

The author(s) declare that they have no competing interests.

## Authors' contributions

Lorna Olckers is a Lecturer in the School of Public Health and Family Medicine, and is presently the Course Convenor for Interprofessional Learning in the Faculty of Health Sciences.

Trevor Gibbs was previously Director of the Education Development Unit and Chair of Medical Education at the University of Cape Town, Faculty of Health Sciences. He took the lead on curriculum reform within the Faculty and helped develop the programme of interprofessional education. He is now Educational Consultant to the Association for Medical Education in Europe.

Madeline Duncan is a Senior Lecturer in Occupational Therapy at UCT, and was an active member of the design team and key developer in interprofessional learning within the Faculty of Health Sciences.

All authors had equal input into the development and writing of this paper.

## Pre-publication history

The pre-publication history for this paper can be accessed here:



## References

[B1] ABIM Foundation (2002). Medical professionalism in the new millennium: A Physician Charter. Annals of Internal Medicine.

[B2] LaSala KB, Nelson J (2005). What contributes to professionalism?. Medsurgical Nursing.

[B3] Duncan M, Alperstein M, Mayers P, Olckers L, Gibbs T (2005). Not just another multi-professional course Part 1: Rationale for a transformative curriculum. Medical Teacher.

[B4] Mayers P, Alperstein M, Duncan M, Olckers L, Gibbs T (2005). Not just another multiprofessional course Part 2: Nuts and bolts of designing a transformed curriculum for multi-professional learning. Medical Teacher.

[B5] Olckers L, Gibbs T, Mayers P, Alperstein M, Duncan M (2006). Early involvement in a multiprofessional course: an integrated approach to the development of personal and interpersonal skills. Education for Primary Care.

[B6] Ngwena C (2000). Substantive equality in South African health care: the limits of law. Medical Law International.

[B7] Cameron N (2003). Physical growth in a transitional economy: the aftermath of South African apartheid. Economic Human Biology.

[B8] Thomas S, Gilson L (2004). Actor management in the development of health financing reform: health insurance in South Africa, 1994–1999. Health Policy Plan.

[B9] World Health Organisation (1978). The Alma-Ata Declaration; primary health care. WHO "Health for All" series.

[B10] Council for Higher Education (CHE) (2001). Towards a New Higher Education Landscape: Meeting the Equity, Quality and Social Development Imperatives of South Africa in the 21^st ^Century. Pretoria South Africa.

[B11] Bossers A, Kernaghan J, Hodgins L, Merla L, O'Connor C, Van Kessel M (1999). Defining and developing professionalism. Canadian Journal of Occupational Therapy.

[B12] Van de Camp K, Vernooij-Dassen MJ, Grol RP, Bottema BJ (2004). How to conceptualise professionalism: a qualitative study. Medical Teacher.

[B13] Lorenzo T, Duncan M, Buchanan H, Alsop A, (Eds.) (2006). Practice and service learning in occupational therapy: enhancing potential in context.

[B14] Meissner O (2004). The traditional healer as part of the primary health care team. The South African Medical Journal.

[B15] Adams A, Barr H, Carpenter J, Cleverly H, Dickinson C (2005). The theory-practice relationship in interprofessional education. Higher Education Academy: Health Sciences and Practice London.

[B16] Adams K, Hean S, Sturgis P, Macleod Clark J (2006). Investigating the factors influencing professional identity of first year health and social care students. Learning in Health and Social Care.

[B17] Freeth D, Hammick M, Koppel I, Reeves S, Barr H (2005). Evaluating Interprofessional Education: a self help guide. Higher Education Academy Learning and Teaching Support Network for Health Sciences and Practice London.

[B18] Niemi PM (1997). Medical students' professional identity: self-reflection during the preclinical years. Medical Education.

[B19] Harden RM, Crosby JR, Davis MH, Friedman M (1999). AMEE Guide No 14: Outcome-based education: Part 5- from competency to meta-competency: a model for the specification of learning outcomes. Medical Teacher.

[B20] (2005). Oxford English Dictionary.

[B21] Bates AW, Pool G (2003). Change and stability in teaching with technology. Effective Teaching with Technology in Higher Education: Foundations for Success.

[B22] Stubbings L, Scott JM (2004). NHS workforce issues: implications for future practice. Journal of Health, Organisation and Management.

[B23] Crutcher RA, Then K, Edwards A, Taylor K, Norton P (2004). Multi-professional education in diabetes. Medical Teacher.

[B24] McNeill D, Kelly E (2005). How the national healthcare quality and disparities reports can catalyse quality improvement. Medical Care.

[B25] Williams B (2001). Developing critical reflection for professional practice through problem-based learning. Journal of Advanced Nursing.

[B26] Barr J (2003). Practice-based interprofessional learning. Journal of Interprofessional Care.

[B27] Tait AR (2004). Clinical governance in primary care: a literature review. Journal of Clinical Nursing.

[B28] Rogers CR (1975). Empathic: an unappreciated way of being. Counselling Psychologist.

[B29] Peloquin SM (1995). The fullness of empathy: reflections and illustrations. American Journal of Occupational Therapy.

[B30] Sevenhuijsen S (2003). Principle ethics and the ethic of care: can they go together?. Social Work/Maatskaplike Werk.

[B31] Boelen C (2004). Building a socially accountable health professions school: towards unity for health. Education for Health.

[B32] General Medical Council (2004). Keeping up to date: the GMC guidance on continuing professional development.

[B33] Butler J (1996). Professional development: Practice as text, reflections as process, and self as locus. Australian Journal of Education.

[B34] Schön DA (1987). Educating the reflective practitioner.

[B35] Barnett R (2000). Realising the University in an age of supercomplexity.

[B36] Ramsden P (1992). Learning to teach in higher education.

[B37] Hager P, Gonczi A, Athanasou J (1993). The development of competency-based assessment strategies for professions National Office of Overseas Recognition research paper No8 DEET.

